# Antiviral activity of glucosylceramides isolated from *Fusarium oxysporum* against *Tobacco mosaic viru*s infection

**DOI:** 10.1371/journal.pone.0242887

**Published:** 2020-11-25

**Authors:** Mariana C. Bernardino, Michel Leon C. O. Couto, Maite F. S. Vaslin, Eliana Barreto-Bergter

**Affiliations:** 1 Departamento de Microbiologia Geral, Instituto de Microbiologia Paulo de Góes (IMPG), Universidade Federal do Rio de Janeiro, Rio de Janeiro, Brazil; 2 Departamento de Virologia, Instituto de Microbiologia Paulo de Góes (IMPG), Universidade Federal do Rio de Janeiro, Rio de Janeiro, Brazil; Institute of medical research and medicinal plant studies, CAMEROON

## Abstract

Natural elicitors derived from pathogenic microorganisms represent an ecologic strategy to achieve resistance in plants against diseases. Glucosylceramides (GlcCer) are classified as neutral glycosphingolipids. GlcCer were isolated and purified from *Fusarium oxysporum* mycelium. *F*. *oxysporum* is a plant pathogenic fungus, abundant in soil and causing severe losses in economically important crops such as corn, tobacco, banana, cotton and passion fruit. In this study we evaluate the capacity of GlcCer in inducing resistance in *N*. *tabacum* cv Xanthi plants against *Tobacco mosaic virus* (TMV). Spraying tobacco plants with GlcCer before virus infection reduced the incidence of necrotic lesions caused by TMV. In addition, plants already infected with the virus showed a reduction in hypersensitive response (HR) lesions after GlcCer treatment, suggesting an antiviral effect of GlcCer. Our investigations showed that GlcCer stimulates the early accumulation of H_2_O_2_ and superoxide radicals. In addition, the expression of PR-1 (pathogenesis-related 1, with suggested antifungal action), PR-2 (β-1,3-glucanase), PR-3 (Chitinase), PR-5 (Osmotin), PAL (Phenylalanine ammonia-lyase), LOX (Lipoxygenase) and POX (Peroxidase) genes was highly induced after treatment of tobacco plants with GlcCer and induction levels remained high throughout a period of 6 to 120 hours. Our experiments demonstrate that GlcCer induces resistance in tobacco plants against infection by TMV.

## Introduction

The genus *Fusarium* shows a wide global distribution and is found in both temperate and tropical regions [1]. *Fusarium oxysporum* is a phytopathogenic fungus that is abundant in the soil and causes significant economic losses in crops like corn, banana, cotton and passion fruit. Species belonging to the *F*. *oxysporum* complex (FOSC) can affect a wide range of plants, including annual and perennial dicotyledons and monocotyledons [2, 3]. Field losses associate to FOSC have a high impact and rank 5th in a list of the top 10 plant pathogens of scientific / economic importance [4].

Cerebrosides or monohexosylceramides (CMH) are neutral glycosphingolipids that contain a monosaccharide, usually glucose or galactose, in 1-ortho-betaglycosidic linkage with a ceramide. Studies show that cerebrosides are capable of conferring tolerance to injuries caused by cold in wheat roots by modulating the lipid composition of the plasma membrane [5]. In addition, Umemura *et al*. [6] and Naveen *et al*. [7] also report *Fusarium* cerebroside as an important elicitor, since it confers resistance to several plant species that are affected by fusariosis. Despite important discoveries by both groups, there are still few publications investigating the role of the cerebroside as an elicitor and none of them report an antiviral effect associate to it elicitation.

Systemic acquired resistance, known as SAR, is a broad-spectrum long-lasting defense where infection by a pathogen at a primary site can induce the defense of distal tissues against various types of pathogens. The inducible defense mechanisms that are activated upon pathogen attack include reinforcement of the cell wall involving callose deposition, generation of reactive oxygen species (ROS), accumulation of secondary metabolites like, and phenolic compounds, and the production of so-called pathogenesis-related (PR) proteins [8]. Reimer-Michalski and Conrath [9] report that SAR is a great promise for sustainable agriculture because plant immune responses have broad-spectrum activity lasting for days or months and do not considerably affect plant fitness.

*Tobacco mosaic virus* (TMV), a notorious plant pathogen that caused serious economic losses worldwide, has a wide host range consisting of more than 885 plant species belonging to 65 families [10]. Plants infected by TMV may present leaves mosaic or mottling, necrosis, stunting, leaf curling, and yellowing of plant tissues. The stunting is usually related to infection occurring early during the planting season and leads to great economic losses. TMV shows worldwide distribution in temperate and tropical regions. There are no known vectors and transmission occurs by mechanical contact. The virus can remain in plant residues, soil and water for long periods. A few effective antiviral agents against TMV and other plant viruses were described [11], despite intense research using synthetic and natural compounds looking for this goal [12]. Between 2012–2015, around 264 compounds were tested aiming to achieve anti-TMV protection, including seven from fungi origin [12].

Our study aimed to evaluate the effect of GlcCer species isolated and purified from the *F*. *oxysporum* cell wall on the induction of resistance against TMV infection in *Nicotiana tabacum* cv Xanthi plants. To our knowledge, this is the first report to investigate the role of the cerebroside in inducing resistance to viral plant diseases.

## Methods

### Microorganism and culture conditions

*Fusarium oxysporum* (IOC 4247) was kindly supplied by Dr. Maria Inês Sarquis from FIOCRUZ Fungi Culture Collection and was maintained in Sabouraud modified medium (SAB-M, 2% glucose, 1% peptone and 5% yeast extract). Cell mass was obtained by growing the fungus in Sab-M medium with shaking for seven days at room temperature.

### Extraction and purification of glycosphingolipids from *F*. *oxysporum*

Total lipids from *F*. *oxysporum* mycelia grown in SAB-M were successively extracted at room temperature using chloroform/methanol 2:1 (v/v) and 1:2 (v/v), as described by Vieira *et al*. [13]. Crude lipid extract was partitioned with chloroform/methanol/0.75% KCl (8:4:3 v/v), as described by Folch *et al*. [14]. The lower Folch layer containing neutral lipids was fractionated on a silica gel column which was eluted sequentially with chloroform, acetone and methanol. Glycosphingolipid-containing fractions were loaded on a new silica gel column and eluted with chloroform/methanol with increasing polarity (90:10, 80:20, 70:30 and 50:50) [15]. The glycosphingolipids recovered after purification were monitored by thin layer chromatography (TLC) on silica gel 60 plates developed with chloroform/methanol/2M ammonium hydroxide, 40:10:1 (v/v). The spots were visualized with iodine and orcinol/H_2_SO_4_ [16, 17].

### Sugar analysis

In order to analyze the monosaccharide components, GlcCer species were hydrolyzed with 3 M trifluoroacetic acid at 100°C for 3 h, as described by Calixto *et al*. [18]. Monosaccharides were detected by thin layer chromatography in n-butanol/acetone/water (4:5:1 v/v/v) and visualized using the orcinol-sulfuric acid reagent [18]. Sugars were identified by comigration with reference sugars (rhamnose, glucose, mannose and galactose).

### ESI-MS analysis

GlcCer species were analyzed by electrospray ionization (ESI-MS) in positive (ESI+) ion mode, using an ESI-ion Trap instrument (Model Amazon SL, Bruker, Germany). GlcCer species were dissolved in chloroform/methanol/water (5:4:1 v/v), containing 1 mM lithium chloride and analyzed via direct injection using a microsyringe pump (Hamilton) [18]. Nitrogen was used as nebulizer and carrier gas.

### Plant culture

Seeds of *Nicotiana tabacum* cv. Xanthi were germinated in Top Garden Floreira substrate® supplemented with vermiculite (1:1) and kept in a greenhouse with temperature control (25°C +/- 2°C) for 30 days. After germination, the seedlings were transferred to individual pots containing the same substrate and vermiculite until grown into plants with six true leaves which were used in the experiments.

### Viral inoculum

Viral inoculum was prepared from *Nicotiana tabacum* SR1 leaves previously infected with TMV and stored at -80°C. About 1g of frozen leaves was macerated with 10 ml of phosphate buffer 0.01M pH 7.0. The macerate was them diluted 10 times in phosphate buffer 0.01M pH 7.0 and the resulting viral suspension was mechanically inoculated in three fully expanded leaves per plant.

### Evaluation of the GlcCer species protective and inactivating effects

A high pressure device (W550, Wagner) was used to assist in the disruption of cells by inserting GlcCer species into the leaves, as described by [19], 30 days old plants of *N*. *tabacum* Xanthi with at least six true leaves were sprayed in a greenhouse with phosphate buffer (0,01M pH 7.0) containing 100 μg.ml^-1^ of GlcCer species (ten plants) or phosphate buffer alone (ten plants). After 24 h, three leaves from each plant in the two groups were mechanically inoculated with TMV. As viral infection positive control, an additional group of five non-GlcCer species sprayed plants were mechanically infected with TMV.

To assess whether the position or age of the leaves can influence the protective effect of GlcCer species against TMV, two sets of experiments were performed on a total of 20 plants each. In one set, leaves in positions 1, 2 and 3 of ten GlcCer-treated plants and ten buffer-treated plants treated were inoculated with TMV and, in a second experiment set, inoculation with TMV was performed on leaves in positions 4, 5 and 6 from another set of ten GlcCer-treated and ten buffer-treated plants. The experiments were repeated 3 times with leaves 1, 2 and 3 and 2 times with leaves 3, 4 and 5 and the values obtained were showed as averaged.

The effect of GlcCer species treatment in plants previously infected with TMV was evaluated. TMV was mechanically applied to the surface of *N*. *tabacum* leaves. After 24 hours, 12 independent plants were sprayed with 100 μg.ml-1 of GlcCer:buffer solution and the same number of plants was sprayed with phosphate buffer as negative control.

In order to analyze whether GlcCer species can inactivate virus particles, TMV-enriched extracts prepared as described below were incubated with GlcCer (500 μg.ml^−1^) for 1 h at 25°C. After that period, the GlcCer species containing virus suspension was mechanically inoculated in three leaves of ten tobacco plants.

### Viral quantification

PathoScreen® (Agdia) ELISA kit for specific detection of viruses of the tobamovirus family including TMV was used to identify the presence of TMV in infected plants following Agdia protocol. To quantify the virus, a standard curve was obtained by serial dilution of a 2.0 x 10^7^ TMV particles/g of leaf sample. Leaf samples of GlcCer- and buffer-treated plants inoculated with TMV were collect 72 h after virus infection. Samples were diluted 1:10 on extraction buffer (Adgia Co.).

### Transmission electronic microscopy

To investigate the effect of GlcCer species on virus particles, transmission electron microscopy was performed according to the methodology described by Zhang *et al*. [10] with some modifications as described below. To prepare the virus sample, 2 g of leaves of *N*. *tabacum* SR1 infected with TMV were macerated with 15 ml of 0.01mM potassium phosphate buffer, pH 7. In order to remove leaf debris and other contaminants, sample was centrifuged in Centrifuge Minispin Eppendorf twice at 12,000 rpm for 5 min, with a F-45-12-11 rotor. The supernatant was filtered through a 22 μm pore filter. In order to precipitate the TMV, an ultracentrifugation was performed at 40,000 rpm for 1 h and 30 min, at 10° C, with a SW40 rotor. The precipitate containing the virus was resuspended in 1 ml of 0,01mM potassium phosphate buffer. The TMV solution was mixed with an equal volume of GlcCer (500 μg.ml ^-1^) and incubated for 1 h at 25° C. After incubation, the GlcCer- treated virus particles were observed by transmission electron microscopy using negative contrast with uranyl acetate on a Tecnai G2 F20 FEG microscope (FEI Company, Eindhoven, The Netherlands). TMV incubated only with buffer was used as control.

### Evaluation of disease severity

TMV infection in *N*. *tabacum* cv. Xanthi induces the formation of necrotic lesions characteristic of hypersensitive response (HR) in the inoculated leaves. TMV-induced HR lesions on plant leaves were identified by visual inspection and counted 72 h after TMV infection.

### Statistical analysis

Statistical analysis of all experiments was performed using the Student T test. Data were plotted using GraphPad Prism v5.0 (http://www.graphpad.com) to generate the graphs.

### Determination of total phenolic content

The total phenolic content of the aqueous extracts was evaluated using the Folin-Ciocalteau reagent [20] with modifications. *N*. *tabacum* leaves were elicited with GlcCer species and collected at different times. The aqueous extracts were obtained by macerating the leaves in distilled water with the aid of a porcelain mortar and pestle, followed by filtration and lyophilization. One-hundred milligrams of the lyophilized extracts were reconstituted in 1 ml of 70% aqueous acetone. In a 96-well microtiter plate, 12.5 μl of the extracts and 25 μl of Folin–Ciocalteu reagent were added to each well. Finally, 187.5 μl distilled water and 25 μl of Na_2_CO_3_ (20% in water) were added to the wells and the reaction was incubated at room temperature in the dark for 1 h, after which the results were read at 660 nm in an ELISA reader.

A calibration curve using gallic acid at concentrations varying from 10 to 500 mg.ml^-1^ was obtained and the total phenolic content of the extracts was expressed as gallic acid equivalents (EAG) in mg per g of lyophilized extract.

### Histochemical visualization of hydrogen peroxide and superoxide radical production

For the evaluation of the hydrogen peroxide and superoxide radical production after treatment with GlcCer species, leaves of *N*. *tabacum* cv Xanthi were collected after 30 min, 1, 3 and 6 h of GlcCer incubation. The histochemical visualization method described by Wang *et al*., 2016 was used with modifications. Hydrogen peroxide production was detected using a solution containing diaminobenzidine (DAB) at the concentration of 1 mg.ml^-1^, and superoxide radicals were detected using, a solution containing the nitro blue tetrazolium reagent (NBT) at a concentration of 0.5mg.ml^-1^. The leaves were dipped in Petri dishes containing either solution and kept in a vacuum chamber (400 bar) in the dark for one hour. The leaves were then boiled in absolute ethanol for chlorophyll removal to provide a better visualization of the precipitates formed by DAB or NBT.

### RNA extraction

Leave samples of *N*. *tabacum* cv. Xanthi were collected at different times after 100 μg.ml^-1^ GlcCer species or 0.01M phosphate buffer treatments and grouped in two pools of ten plants each per treatment and time. Samples were immediately frozen in liquid nitrogen and stored at -80°C until use. For RNA extraction, leaves were crushed in liquid nitrogen until the formation of a powder that was transferred to an Eppendorf tube and maintained at -80°C for subsequent extraction of total RNA.

The total RNA was extracted using Trizol (Thermo Fisher Scientific Co.) according to the manufacturer's instructions. Concentration and purity were determined using a NanoDrop2000 spectrophotometer (Thermo Fisher Scientific Co.). RNA integrity was verified by 0.8% agarose gel electrophoresis in tris acetate buffer (0.5x) followed by ethidium bromide staining. RNA samples were treated with RQ1 RNase-Free Dnase (Promega Co.) before reverse transcription reactions.

### Reverse transcriptase reaction and real-time quantitative PCR

Synthesis of complementary DNA (cDNA) was made using 1 μg of total RNA, SuperScript IV Reverse Transcriptase (Thermo Fisher Scientific Co.) and 100 μM of Oligo dT primer. After cDNA synthesis, the samples were diluted 25 times in MilliQ water.

RT-qPCR reactions were performed in 96- wells plates containing per well 2.5 μl of cDNA, 6 μL of SYBR Green/ROX qPCR Master Mix (Thermo Fisher Scientific), 0.6 μl of forward and reverse primers (10 mM) of each gene analyzed and MilliQ water to a final volume of 25 μl. The reactions were carried out in a 7500 Fast Real-Time PCR System (Applied Biosystems). The analyses were made from the average of a technical triplicate from each biological replicate, and the relative expression was calculated by the method 2^-ΔΔCt^ following as proposed by Livak and Schmitgen [21]. Primers used for qPCR analysis were described by Mattos *et al*., 2018. Annealing temperature of all genes evaluated was 60°C, except for *PAL* and *PR5*, whose annealing temperature was 62°C. *PR-1* (pathogenesis related protein-1 with suggested antifungal action), *PR-2* (β-1,3-glucanase), *PR-3* (chitinase), *PR-5* (osmotin/thaumatin-like), *PAL* (phenylalanine ammonia-lyase), *LOX* (lipoxygenase) and *POX* (Peroxidase) genes expression was evaluated. PP2A (phosphatase 2A) and Ntbuc (ubiquitin) were used to normalize cDNA expression levels [22].

### Effect of GlcCer species on plant development

To evaluate the effect of GlcCer species treatment on *N*. *tabacum* cv Xanthi, 30 plants with an equal number of leaves and comparable size were selected. Of these, ten plants were left untreated, ten were treated with 0.01M potassium phosphate buffer and another ten plants were treated with GlcCer species at a concentration of 100 μg.ml^-1^. The plants were kept in a greenhouse and 30 days after theirs sizes, without considering the roots, were measured in cm.

## Results

### Extraction and purification of GlcCer species

**Fig 1:**
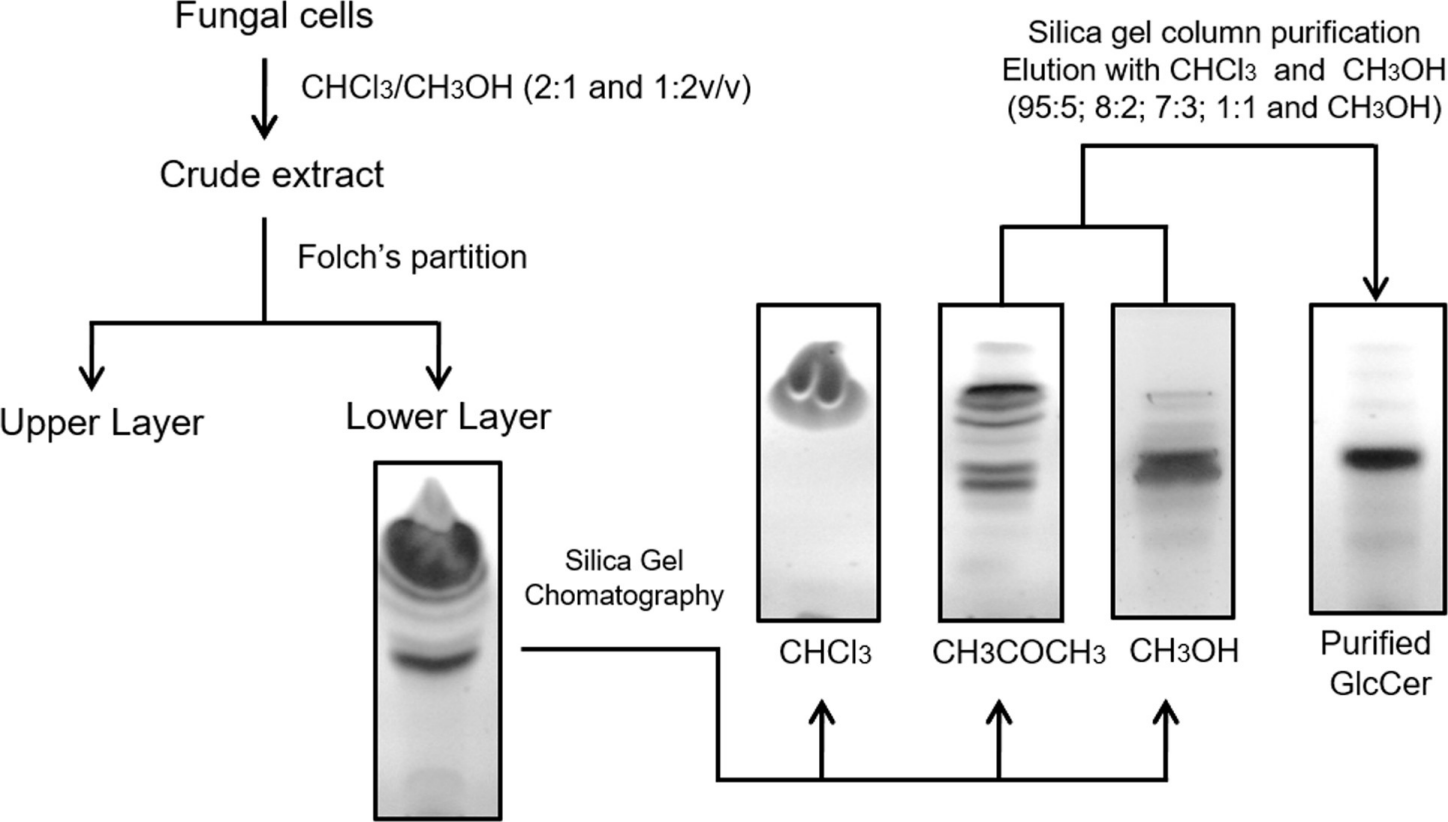
Scheme of the isolation and purification of glucosylceramides from *F*. *oxysporum*.

GlcCer species was extracted with chloroform and methanol in the proportions 2:1 and then with 1:2 (v/v). The crude extract was fractionated according to Folch et al. [14]. The lower phase lipids were fractionated on a silica gel column eluted with chloroform, acetone and methanol. GlcCer species was predominantly present in the acetone fraction and was purified by successive chromatography on silica gel. For further purification, it was necessary to elute the column with chloroform and methanol in different concentrations, as shown in Fig 1. The purified component was eluted with chloroform/methanol 8:2 and 7:3 (v/v). Confirmation of the presence of the substance at different stages of purification was monitored by thin layer chromatography (TLC).

### Chemical characterization of *F*. *oxysporum* GlcCer species

**Fig 2:**
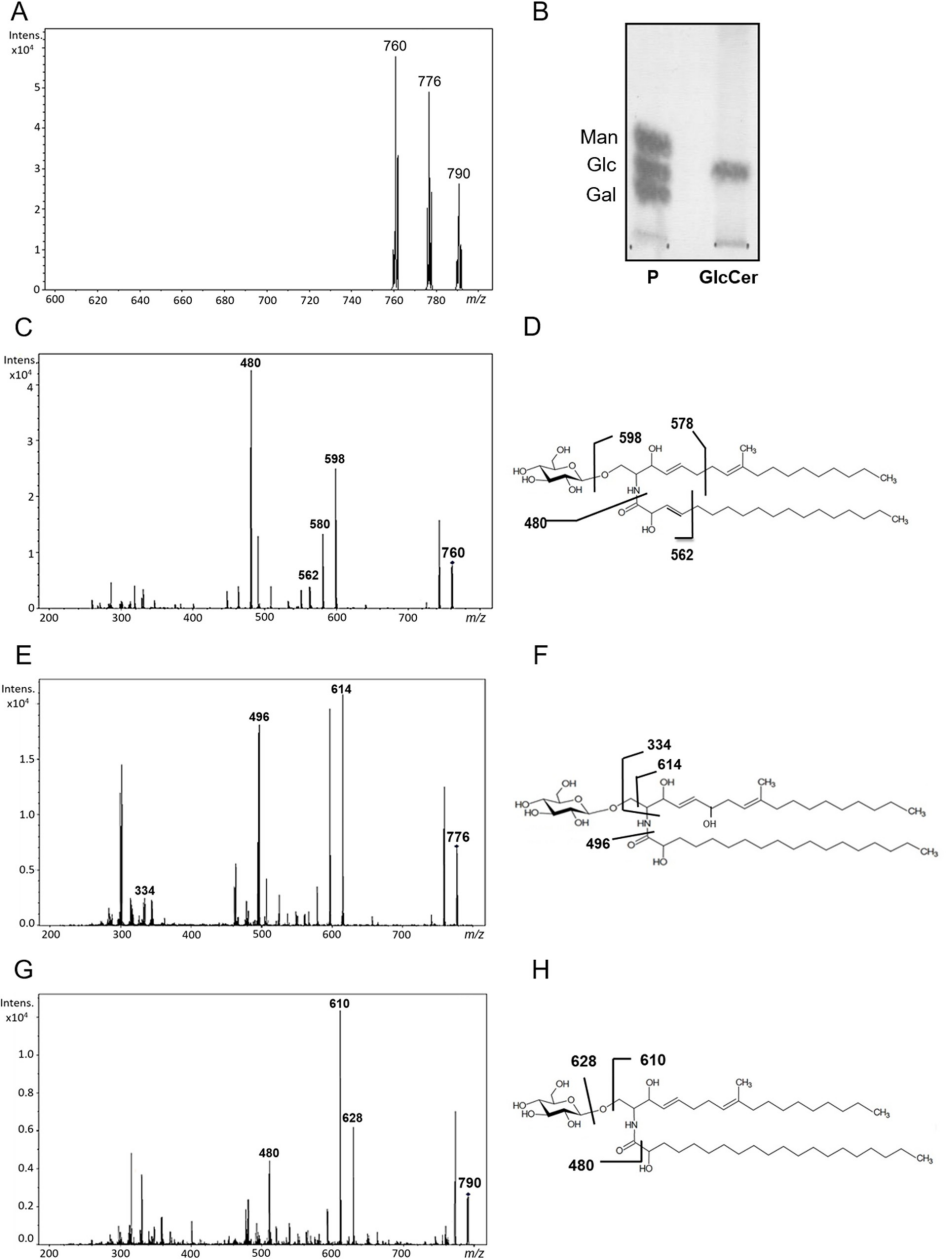
ESI-MS (positive ion mode, Li+ adducts) analysis of the GlcCer species of *F*. *oxysporum*. (A) MS1 spectrum. (C, E and G) ESI-MS2 of the ions species *m/z* 760, 776 and 790, observed in (A) and proposed structures for the major GlcCer species in *F*. *oxysporum* (D, F and H). (B) HPTLC plate of monosaccharides from *F*. *oxysporum* CMH. 1. Galactose, glucose and mannose standards; 2. Glucose from CMH. The sugars were detected using the orcinol-sulfuric acid spray reagent.

Purified GlcCer species was submitted to mass spectrometry using electrospray ionization (ESI-MS), revealing three molecular ions with *m/z* [M + Li] 760, 776 and 790 (Fig 2A). These ions were subjected to a second fragmentation (MS2), generating smaller fragments, such as *m/z* 598.5, *m/z* 614.5 and *m/z* 628.5, which correspond to the ceramide portion of the molecular ions *m/z* 760, 776 and 790, respectively (Fig 2C, 2E and 2G). The glycosphingolipids presenting *m/z* 760, 776 and 790 were identified as N-2'-hydroxyoctadecenoic-1-β-D-glucopyranosyl-9-methyl-4,8-sphingadienine (Fig 2D), N-2'-hydroxyoctadecanoic-1-β-D-glucopyranosyl-9-methyl-4-hydroxy-4,8- sphingadienine (Fig 2F) and N-2'-hydroxyeicosanoyl-1-β-D-glucopyranosyl-9-methyl-4,8-sphingadienine (Fig 2G), respectively. Glucose was identified as the monosaccharide component of *F*. *oxysporum* GlcCer species after hydrolysis and TLC analysis (Fig 2B).

### GlcCer species induces a protective effect against TMV infection in tobacco plants

**Fig 3:**
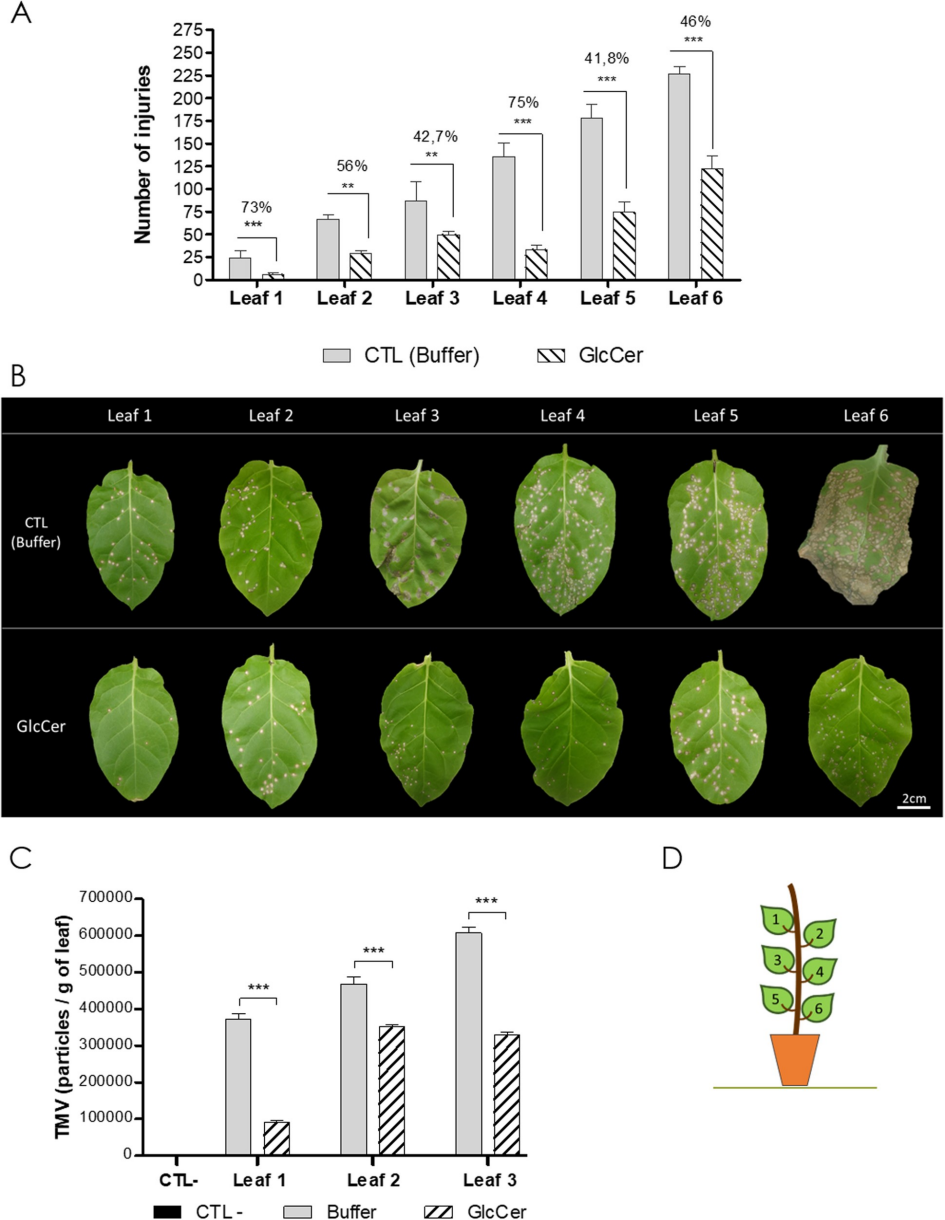
(A) Number of necrotic lesions observed in *Nicotiana tabacum* cv. Xanthi plants elicited with GlcCer species or buffer (CTL) and mechanically inoculated with TMV 24h after treatment. Necrotic lesions were count 72h after virus inoculum. (B) Representative image of necrotic lesions in *N*. *Tabacum* cv Xanthi of all leaves evaluated after 72h of TMV inoculum. The control (CTL Buffer) were leaves treated only with phosphate buffer (upper line), while in the lower line, leaves were treated with 100 μg.ml^-1^ of GlcCer. (C) TMV titer in infected plants treated with GlcCer species or buffer 72h before TMV inoculation. (D) Scheme of the position of the evaluated leaves. Asterisks denote values statistically different from controls **P <0.01, ***P <0.001. ***P <0.0001.

As fungus cerebroside GlcCer species was reported previously to elicit protection against fungus attack in plants, experiments were performed to check if this elicitation could also protect plants against virus infections. *F*. *oxysporum* GlcCer species at a concentration of 100 μg.ml^-1^ was sprayed on 30 days old *Nicotiana tabacum* Xanthi plants under greenhouse conditions, followed by a TMV mechanical inoculation 24 hours after GlcCer species elicitation. The number of TMV-induced necrotic lesions was counted 72 hours after viral inoculum (hpi). A drastic reduction in the number of TMV-induced necrotic lesions was observed in the leaves of GlcCer-treated plants (Fig 3B). The observed reductions were 71% on leaf 1, 56% on leaf 2, 42.7% on leaf 3, 75% on leaf 4, 41.8% on leaf 5 and 46% on leaf 6, respectively, when compared to the control which was sprayed with phosphate buffer (Fig 3A and 3B). Leaves from different positions were infected with TMV in order to observe if leaf position can impact TMV tolerance effect. Younger leaves seem to show a more pronounced response against the virus, with greater reduction in the number of necrotic lesions, than the more developed leaves from positions 5 and 6. Leaves from position 4, unexpectedly showed a higher protection (75%) than leaves from positions 2 and 3. Besides the reduction in the number of necrotic lesions, virus titer also showed a decrease in GlcCer species treated leaves (Fig 3C) by viral quantification ELISA. Fig 3B shows a representative pattern of the HR-necrotic lesions observed in each leaf after buffer and GlcCer treatments. Fig 3D Scheme of the position of the evaluated leaves. These results clearly demonstrate the GlcCer species ability to partially protect plants of *N*. *tabacum* cv Xanthi in 55,75% against TMV infection.

### Evaluation of GlcCer species effect on TMV previously infected plants

**Fig 4:**
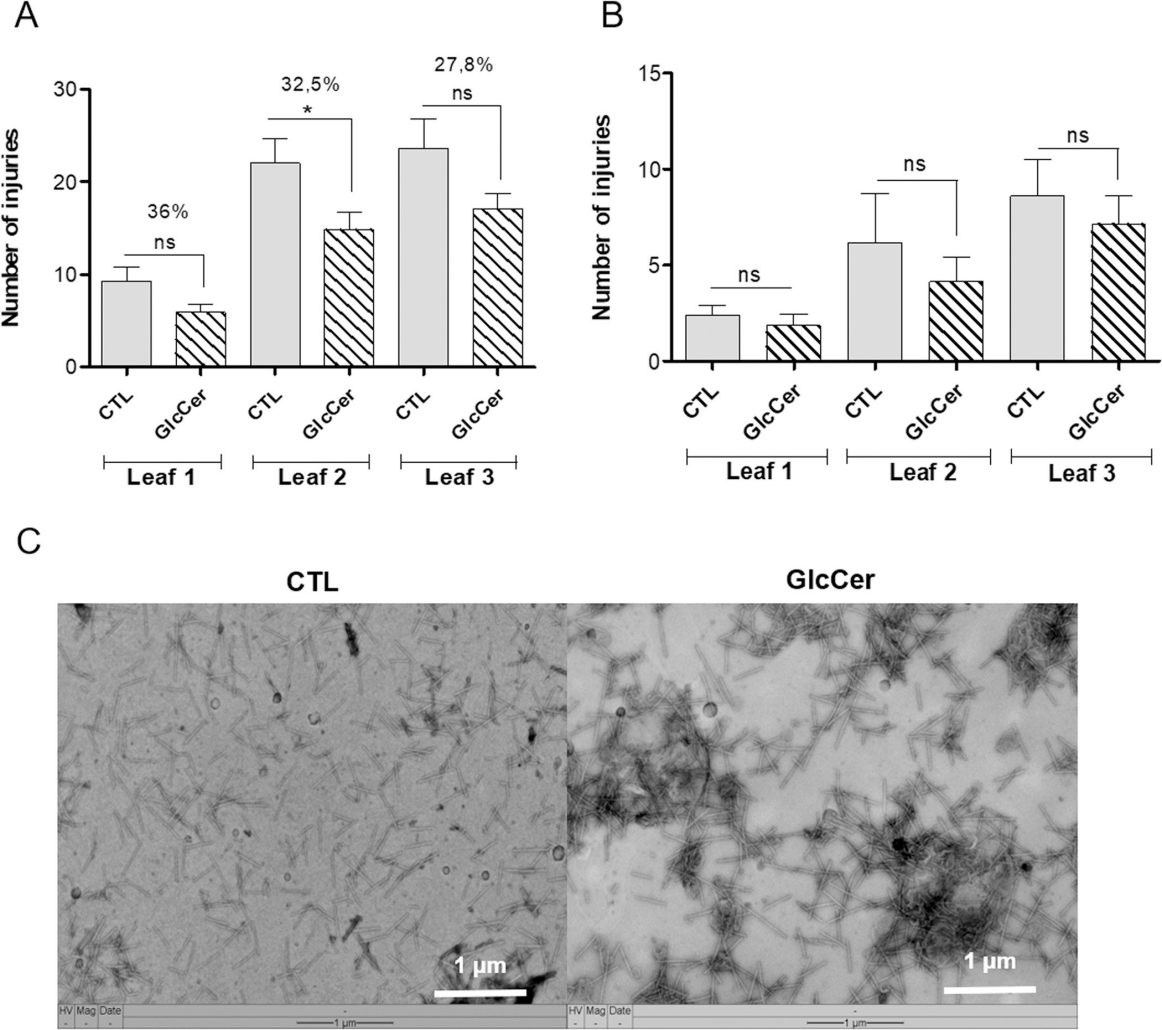
Evaluation of the GlcCer species treatment in previously infected plants and of GlcCer species incubation with TMV in *N*. *tabacum* cv. Xanthi plants. (A) Effect of GlcCer species sprayed on *N*. *tabacum* cv Xanthi 24h after TMV infection on leaves 1, 2 and 3. (B) TMV infectivity of TMV particles previously incubated with GlcCer species for 1h and inoculated in *N*. *tabacum* cv Xanthi in leaves 1, 2 and 3. (C) Transmission electron microscopy of untreated TMV particles (CTL) and TMV particles treated with GlcCer species from *F*. *oxysporum*. Bars = 1μm. Values represent the mean ± SD of ten independent experiments. ns = no significant difference. Asterisks denote values statistically different from control. *P = 0,0386.

Our previous experiments showed that GlcCer species can protect tobacco plants against subsequent viral infections. However, treatment with GlcCer species is not as effective in helping infected plants to eliminate the virus when the infection is already established. The tobacco plants were mechanically inoculated with TMV and 24 hours later sprayed with 100 μg.ml^-1^ of GlcCer species. It was observed that post-infection treatment with GlcCer species decreased the number of necrotic lesions by TMV on leaves in positions 1, 2 and 3 by 36%, 32.5% and 27.8%, respectively, when compared to the control (Fig 4A), showing that treatment with GlcCer species is able to reduce the number of lesions when the plant was already infected.

### Effect of pre-treatment of TMV with GlcCer species

In order to understand if GlcCer species is helping the plant to eliminate the virus by either strengthening its immune system or by inactivation of the virus in an infected plant, TMV was incubated *in vitro* with *F*. *oxysporum* GlcCer species for one hour and subsequently mechanically inoculated in *N*. *tabacum* Xanthi plants. No significant difference in the number of necrotic lesions was observed on plants that received TMV virus particles previously incubated with GlcCer species when compared to the control (Fig 4B).

After incubation, virus was observed by transmission electron microscopy. Microscopy showed that TMV particles treated with GlcCer species apparently did not suffer visible morphological alterations. The shape of most TMV particles remained intact, but we observed a particle aggregation phenomenon (Fig 4C). The aggregation of TMV particles observed after in vitro incubation of the virus with GlcCer species apparently did not interfere in virus infectivity as no differences were observed between the number of necrotic lesions between the treatment.

### Evaluation of total polyphenol content after GlcCer species treatment

**Fig 5:**
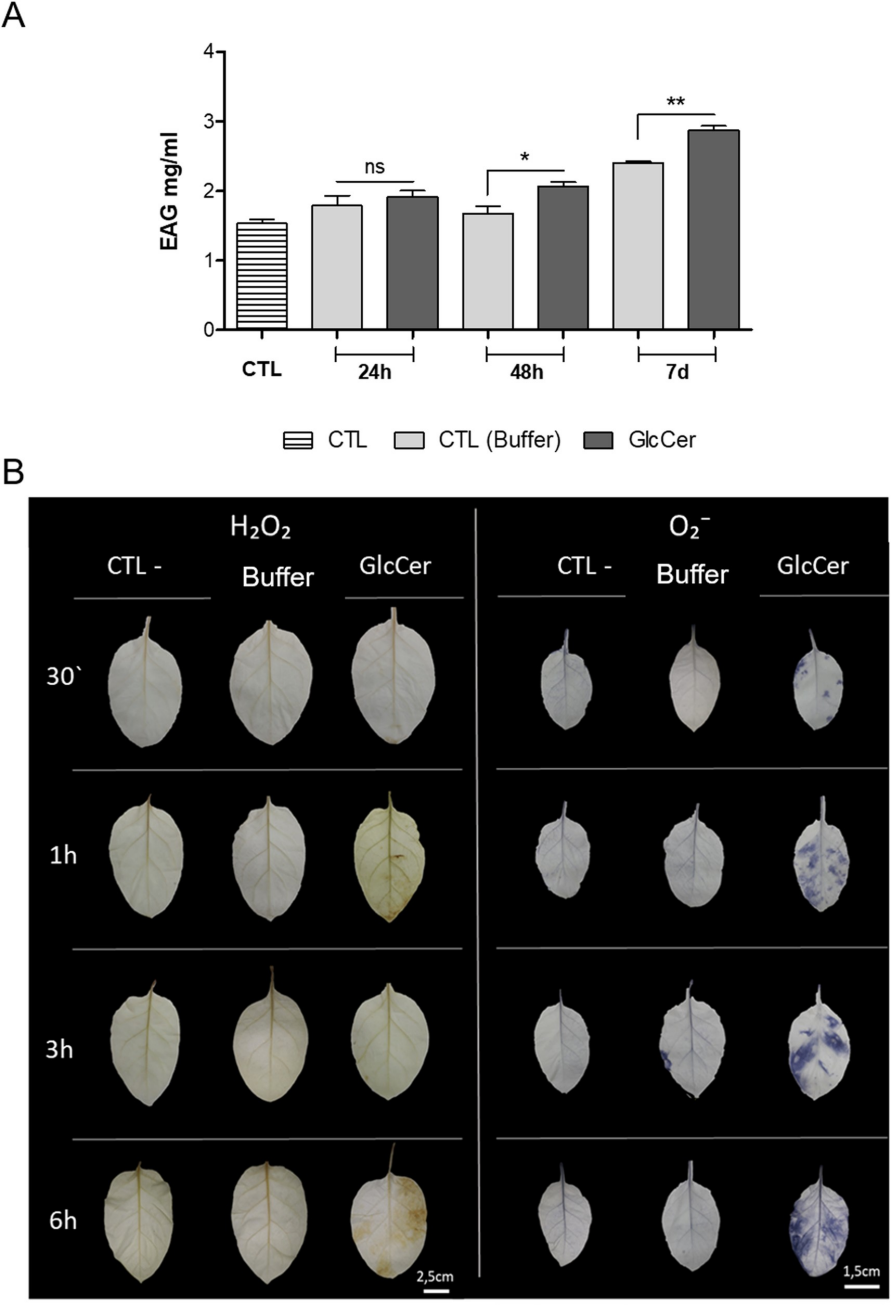
(A) Total polyphenol content of aqueous extracts of *N*. *Tabacum* cv Xanthi treated with GlcCer species (100μg.ml^-1^) and collected at 24 and 48h and 7 days after elicitation (24h, 48h and 7 days). (B) Histochemical visualization of the accumulation of reactive oxygen species (ROS) in leaves of *Nicotiana tabacum* cv. Xanthi collected at different times after treatment with GlcCer species of *F*. *oxysporum*. On the left, the accumulation of H_2_O_2,_ is evidenced by the brown coloration (DAB precipitation). On the right, the accumulation of O₂⁻, is evidenced by the blue color (NBT precipitation). Values represent the mean ± SD of three different plants. ns = no significant difference. Asterisks denote values statistically different. *P = 0.036 and **P = 0.022.

The pathway of phenolic metabolism is responsible for the production of several secondary metabolic compounds that strengthen plant defenses upon biotic and abiotic stress. To observe if the phenolic pathway is activated by GlcCer species, we determined the content of phenolic compounds of *N*. *tabacum* cv Xanthi after GlcCer species treatment. The total amount of polyphenols in leaf extracts was assessed using the method of Folin-Ciocalteu and expressed as milligrams of gallic acid equivalents per gram. Analyzing total polyphenol levels at different times after treatment with 100μg.ml^-1^ of GlcCer species (Fig 5A) we observed a significant increase of polyphenol synthesis after 48h (23%) and 7 days (19%) when compared with the control plants treated with buffer.

### GlcCer species treatment induces the accumulation of reactive oxygen species (ROS)

One of the first lines of defense in plants is the accumulation of reactive oxygen species (ROS). Histochemical analysis of the accumulation of ROS was performed in GlcCer species treated plants between 0.5–6.0 h after treatment using diaminobenzidine (DAB) to evaluate the presence of H_2_O_2_, or nitroblue tetrazolium (NBT) to evaluate the presence of O_2_^-^ (Fig 5B). The accumulation of H₂O₂ was observed at all time points tested. Moreover, it is interesting to note that the highest levels of H_2_O_2_ accumulation were reached after 6 h. It was also possible to observe that the accumulation of O₂⁻ occurs at all time points tested, with maximum values reached after 3 and 6 h. These results indicate that GlcCer species is capable of inducing ROS production in tobacco plants and activate a classical first line of defense.

### Analysis of expression of plant defense genes

**Fig 6:**
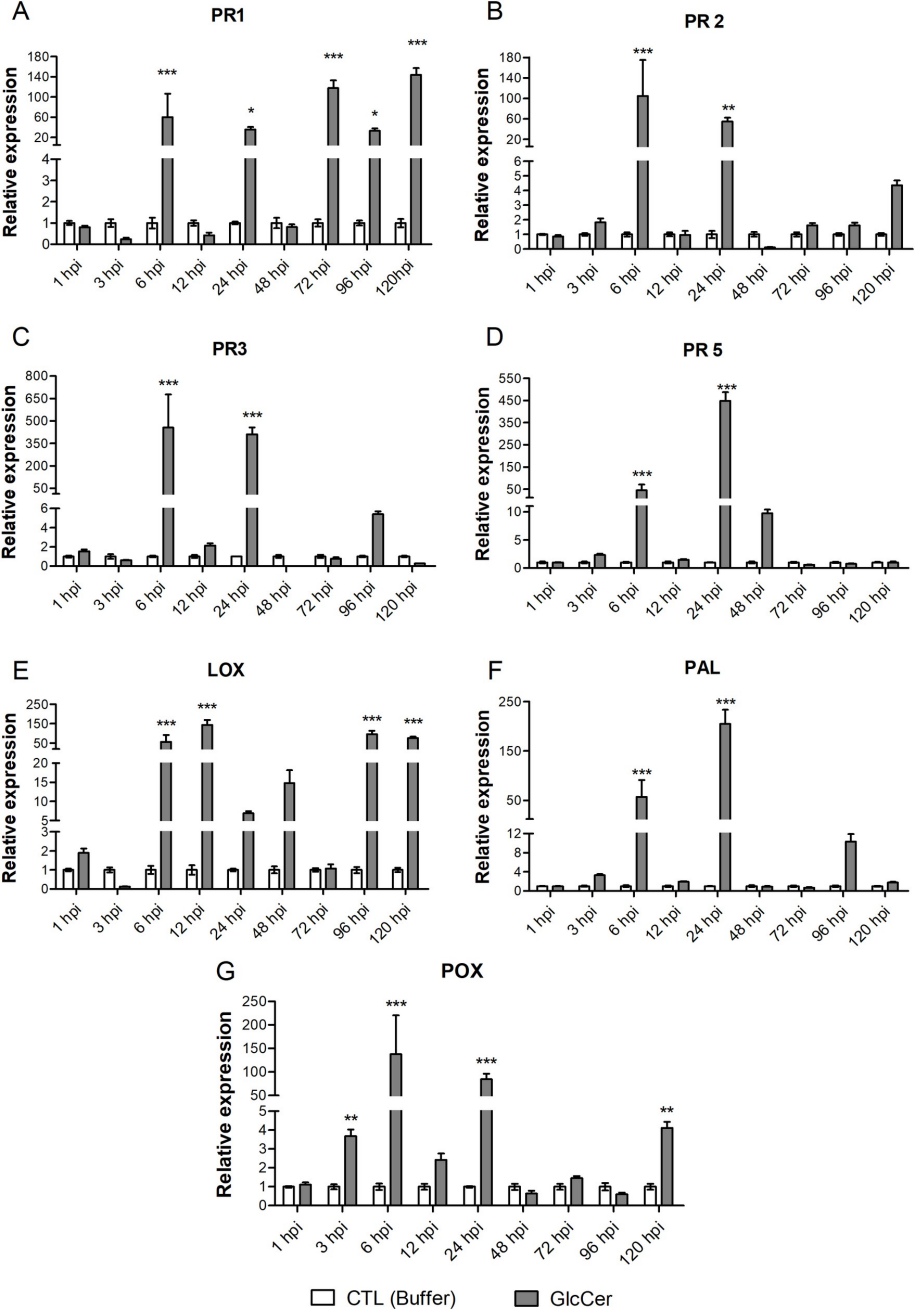
Expression of genes related to the defense of plants of *N*. *tabacum* cv Xanthi treated with GlcCer species of *F*. *oxysporum* at different times. *PR1*: suggested antifungal function, *PR2*: *β*-(1,3) glucanase; *PR3*: Chitinase; *PR5*: Osmotin-like; *POX*: Peroxidase; *LOX*: Lipoxygenase and *PAL*: Phenylalanine ammonia lyase. Constitutive genes used for normalisation: *PP2A* and Ubiquitin. The analyzes were made from the average of two biologically independent experiments and technical triplicate of each biological replicate, and the relative expression was calculated by the 2^-ΔΔCt^ method. Asterisks denote values statistically different from control. *P <0.05, **P <0.01, ***P <0.001.

To evaluate the effect of GlcCer species treatment on SAR induction in *N*. *tabacum* cv Xanthi plants, expression of several defense-related genes was evaluated by real-time PCR between one and 120 h after treatment (Fig 6). All the four PR genes analyzed showed strong induction by GlcCer species. The expression of PR1 gene, a classical SAR marker, started to increase six hours after GlcCer species treatment and kept its expression high during almost all time points, reaching its peak expression after 120 h when it was 147.7 times more expressed than in control plants. The PR2 gene, which encodes a β-(1,3) glucanase and is also considered a SAR marker, reached its highest expression after six hours, being 104.5 times more expressed than in the controls. After 24 h, its expression was still 54.6 fold higher than the control. The PR3 gene, which encodes a chitinase, also had its highest level of expression after six hours (454.8 fold), and remained very high after 24 h (409.6 fold). The thaumatin-like PR5 gene showed a later induction, with the highest peak of expression after 24 hours, being 448.2 times more expressed than in the control. However, its expression decreased over time.

Looking for other classical markers of plant defense activation, we observed that lipoxygenase (LOX) gene had its peak expression after 12 hours, being 143.7 times more expressed than in the control plants, and remained at very high levels of expression during later time points. The peroxidase (POX) gene showed a first expression increase after three hours, being 3.6 times more expressed, reaching its peak expression after six hours, with a 137.6 fold increase. These data agree with data observed in the ROS histochemistry experiment where the higher accumulation of H_2_O_2_ was observed six hours after GlcCer treatment.

The evaluation of the phylalanine-ammonyalyase (PAL) gene which encodes the first enzyme of the phenolic pathway showed that its expression was strongly induced by GlcCer species treatment. PAL transcripts had their first increase in expression after six hours, reaching peak expression 24 h after treatment being 219 times more expressed than in the control.

These results show that treatment with *F*. *oxysporum* GlcCer species was able to induce a strong increase in gene expression over time, especially after six hours, indicating that all analyzed genes are activated early after treatment and SAR is beeing induced.

### GlcCer species do not impair tobacco plants development

**Fig 7:**
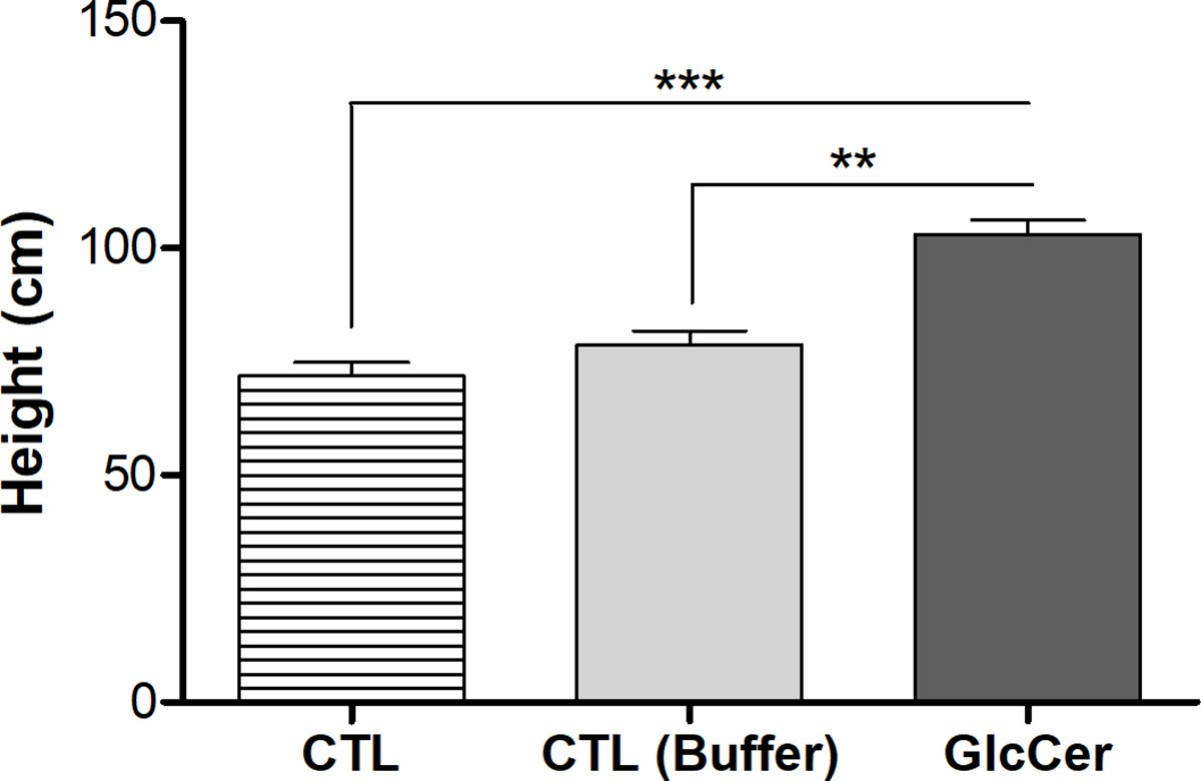
Effects of GlcCer species on plant height. **GlcCer species 100 μg.ml**^**-1**^ **solution was used to spray plants and controls.** CTL–untreated plants, Buffer–plants sprayed with potassium phosphate buffer 0.01M and GlcCer species–plants sprayed with glucosylceramide at a concentration of 100μg.ml^-1^. The measure of the sizes of 10 plants for each treatment were made 30 days after spraying. **P = 0,0014 and ***P = 0,0004.

GlcCer species seems to have a positive effect on plant growth. During experiments in the greenhouse, it was observed that plants sprayed with GlcCer species were healthier and stronger than control plants treated with buffer. Therefore, an experiment was implemented to evaluate if GlcCer species may influence plant height. Height evaluation of GlcCer species treated and untreated plants showed that plants treated with GlcCer species were 23,5% taller than those treated with buffer and 30,3% taller than untreated control plants and, consequently (Fig 7). So, unlike some other antiviral compounds, GlcCer species does not delay plant growth and seems to even improve it.

## Discussion

In order to solve the problem of indiscriminate use of agricultural pesticides researchers from all over the world are looking for new options for the control of pathogens that are efficient, economical and of low environmental impact. A promising alternative is the use of plant defense inducers, alone or in combination with beneficial microorganisms or pesticides [23]. Plant defense bioestimulators, elicitors or activators generally induce a wide range of systemic resistance [24], with a significant reduction in disease symptoms caused by different types of pathogens [25]. The use of mechanisms designed to activate an induced or constitutive defense response in order to prevent plant colonization by phytopathogens is a strategy that has been followed [26]. Elicitor molecules are capable of inducing a defense response in plants, improving immunity and decreasing their susceptibility to diseases caused by phytopathogens [27]. These molecules can be extracted directly from microorganisms or synthesized in laboratory [28]. In 2003, Narasimhan and collaborators demonstrated that the increase in the expression of glycoproteins of the cell wall of *F*. *oxysporum* f. sp. *nicotianae* corresponded to a greater severity in the lesions presented in tobacco leaves [29]. Years before, Koga *et al*. [30] isolated and identified compounds with the elicitor action from the fungus *Magnaporthe grisea*, *a* phytopathogen of rice. These compounds had their structures analyzed and were identified as sphingolipids. Treatment of rice leaves with these cerebrosides was able to induce the production and accumulation of phytoalexins, cell death and increased resistance against compatible pathogens.

In this work, we propose the use of glucosylceramides species (GlcCer) extracted from the mycelium of *F*. *oxysporum* as an elicitor of plant immunity as an alternative method to combat diseases affecting the plants. Three molecular species of GlcCer were identified by mass spectrometry in *F*. *oxysporum* with *m/z* 760, 776 and 790, containing the long chain base, 9-methyl-4,8-sphingadienine, linked by amide bond to 2-hydroxyoctadecenoic acid (C18: 1 OH), 2-hydroxyoctadecanoic acid (C18: OH) and 2-hydroxyeicosanoic acid (C20: OH), respectively. These structures are similar to those found in other fungi [31, 32], such as *A*. *fumigatus*, *C*. *albicans* and *Scedosporium* species, evidencing the conserved characteristic of this molecule in the different fungi studied. Long chain base (9-methyl-4,8-sphingadienine) and hydroxylated fatty acids are present in all of these fungi and the difference between these molecules occurs in relation to the unsaturation and size of the fatty acid chain [31]. Several studies have demonstrated the importance of this molecule in crucial processes for fungal cells, such as cell differentiation, growth and viability [31], what may explain why these glycosphingolipids are highly conserved among different species.

Research shows that other substances have already been used as inducers of resistance against TMV, as is the case of Munronin O, isolated from the *Munronia henryi* Harms plant, that presented curative, protective and inactivating activities of TMV greater than those of ningnanmycin [33]. Zhao and collaborators also showed that the application of fatty acids (oleic acid) can increase resistance against TMV and the antiviral activity can be attributed to activated expressions of a series of defense-related genes as performed in our work [34].

Umemura *et al*. [6] purified cerebroside C from various strains of *Fusarium oxysporum*. This molecule corresponds to N-2'-hydroxyoctadecenoic-1-β-D-glucopyranosyl-9-methyl-4,8-sphingadienine described in our work. Here, however, we characterize also two more cerebroside, N-2'-hydroxyoctadecanoic -1-β-D-glucopyranosyl-9-methyl-4-hydroxy- 4,8-sphingadienine and N-2'-hydroxyeicosanoyl-1-β-D-glucopyranosyl-9-methyl-4,8-sphingadienine. A 2-hydroxyeicosanoic acid (C20: OH) fatty acid is described for the first time in *Fusarium oxysporum* GlcCer isolated in the present work.

Analyzing the impact of the *F*. *oxysporum* cerebroside GlcCer species treatment in tobacco plants, we assessed the effect on the production of phenolic compounds. Our results indicated that GlcCer species can induce the production of phenolic compounds in tobacco plants in a time-dependent manner. Polyphenols are secondary metabolites synthesized along the phenylpropanoid pathway. Polyphenols are found abundantly in a wide variety of foods, such as fruits, vegetables, seeds and cereals, and in beverages, such as wine. They are currently a topic of great scientific attention due to the interest in their potential health benefits. In addition, the class of flavonoids and isoflavonoids can also act as phytoalexins due to their antifungal and antibacterial effect [35].

According to Heller and Tudzynski [36], reactive oxygen species play a fundamental role in the interactions between pathogens and plants and it is one of the first events that occur in the recognition of a pathogen by the plant. Besides its direct antimicrobial effect, ROS generation is also involved in cell signaling associated with the expression of genes related to pathogenesis (PR), hypersensitivity response (HR) and also to the development of SAR [36, 37]. In 2010, Kim and collaborators reported that *A*. *thaliana* plants that overexpressed the RCI3 gene, which encodes peroxidase, had an increase in the production of ROS in the roots when these plants had a low potassium availability [38]. In addition, Choi and collaborators also demonstrated that the overexpression of *A*. *thaliana* CapO2 gene, which encodes an extracellular peroxidase in pepper, increased H_2_O_2_ concentrations in response to infection by the bacterium *Pseudomonas syringae* [39]. To investigate GlcCer species ability to induce ROS synthesis, we evaluated the generation of superoxide radical and hydrogen peroxide and peroxidase gene expression in tobacco plants. Treatment with *F*. *oxysporum* GlcCer species was able to induce the production of O_2_^-^ and H_2_O_2_ as detected by histochemical assays. The largest accumulation occurred after 6 h and coincides with the peak of POX expression, as shown by RT-qPCR.

Umemura *et al*. [6] demonstrated that treatment of lettuce, tomato, melon and sweet potato plants with a fungal cerebroside was able to induce the production of ROS and the expression of PR *genes* protecting these plants against *F*. *oxysporum* infection. In addition, Wang *et al*. [40] demonstrated that the PeBA1 protein extracted from *Bacillus amyloliquefaciens* was able to protect *N*. *tabacum* plants against TMV infection, demonstrating the activity of a molecule of bacterial origin against a viral disease. The results observed here show for the first time that a fungal GlcCer can act as possible antiviral agent in plants. GlcCer species treatment of *N*. *tabacum* cv Xanthi plants induced a minimum of 42.8% reduction in the number of necrotic lesions when compared to the control. A 75% reduction was detected in younger leaves, indicating the anti-viral potential of GlcCer species.

Elicitation of defense in plants generally is associated to the induction of SAR. Among the genes commonly induced during SAR establishment, increased PR1 expression is recognized as one of the main markers of SAR [29, 41]. The silencing of PR1 in *Arabidopsis thaliana* led to a greater susceptibility to *Phytophthora parasitic* oomycete infection while *N*. *tabacum* plants that shown PR1 gene overexpression showed an increased resistance against *Phytophthora parasitic* and *Peronospora tabacina* [42, 43]. Here, it was possible to observe that the *PR1* gene expression is highly induced over various times tested, thus indicating the activation of SAR. The two genes encoding for enzymes with antifungal action such as *PR3* (chitinase) and *PR2* (*β*-(1,3) glucanase), presented a similar expression profile, with increased expression between 6 and 24 h.

As previously mentioned, the establishment of SAR is mediated by the accumulation of salicylic acid (SA) in tissues distal to the site of infection, and this accumulation is responsible for triggering the defense response pathway. In tobacco, the main pathway of salicylic acid synthesis is through the phenylpropanoid pathway, which is mediated by the action of the PAL enzyme [44]. The increased expression of this gene is associated with the rapid accumulation of SA [28]. Our results showed that PAL activity increased rapidly in the treated plants. PAL is an enzyme that catalyzes the first step in the metabolism of phenylpropanoids. PAL activity reached its first peak in the first six hours, followed by a gradual increase with 24 and 96 hours after elicitation.

All plant defense related genes studied in this work showed a strong increase in their expression in the period of up to 6 h after GlcCer species treatment, with exception of the gene encoding peroxidase (*POX*), which had a significant increase earlier, after 3 h. These data suggest not only the establishment of SAR but also indicate the rapid development of this response.

The high expression of almost all genes tested, was detected following a period of basal expression or even repression. We hypothesize that these findings could be correlated with the circadian cycle. NPR1 protein plays an essential role in the transcription of genes related to the circadian cycle and is also involved in the development of plant defense responses and the establishment of SAR [45, 46]. These dynamics between the expression of defense genes and the relationship with the circadian cycle may be related to the results reported above.

Our results are in agreement with those described by Naveen *et al*. [7], where the defense mechanisms of pepper plants (*Capsicum annum*) are induced after treatment with a cerebroside extracted from *Colletotrichum capsici*, a pepper phytopathogen. This molecule was able to induce the initial accumulation of H_2_O_2_ and increased the production of the enzymes PAL, POX and LOX. In addition, it was able to protect the plant against infection by *C*. *capsici*. Studies by Umemura *et al*. [47] also demonstrated that cerebrosides extracted from *Magnaporthe grisea* are involved in the production of phytoalexins and PR proteins in rice leaves. In field experiments, the cerebroside elicitors effectively protected rice plants against the rice blast fungus, an economically devastating agent of disease of rice in Japan. The cerebrosides also protected rice plants from other diseases. These compounds occur in a wide range of different phytopathogens and act as general elicitors in a wide variety of rice-pathogen interactions [47].

According to the results described in this work, it is possible to suggest that *F*. *oxysporum* glucosylceramide is a strong inducer of systemic acquired resistance witch consequently induces a high antiviral status in the treated plants. The protective effect observed after treatment is quite expressive and indicates that this treatment could induces tolerance for other plant infectious virus. New experiments must be performed to test this hypothesis.

## Supporting information

S1 Raw images (PDF)
