# Supplementary material for: Antiviral activity of glucosylceramides isolated from Fusarium oxysporum against Tobacco mosaic virus infection
Source: PLoS One. 2020 Nov 25;15(11):e0242887. doi: 10.1371/journal.pone.0242887 (PMC7688173; doi:10.1371/journal.pone.0242887)
Supplement: S1 Raw images — (PDF) [file pone.0242887.s001.pdf]

## Schematic representation of blots used in Figure 1

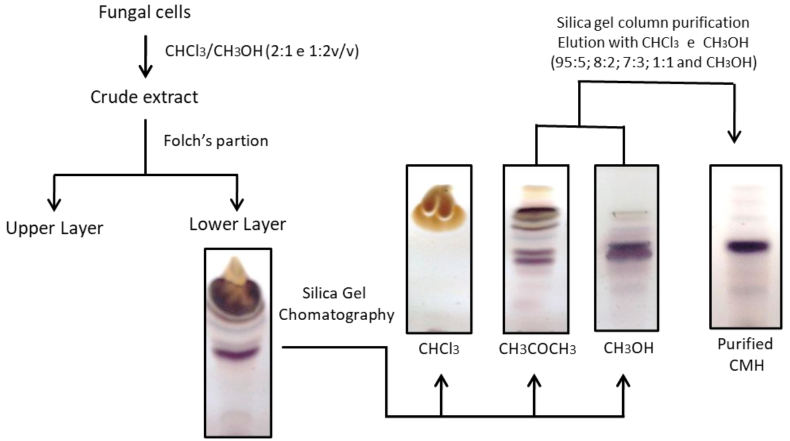


Figure 1

S1A

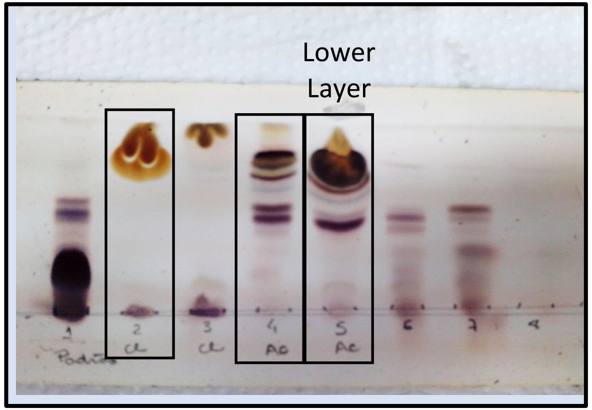


$CH_3Cl$ $CH_3COCH_3$

S1B

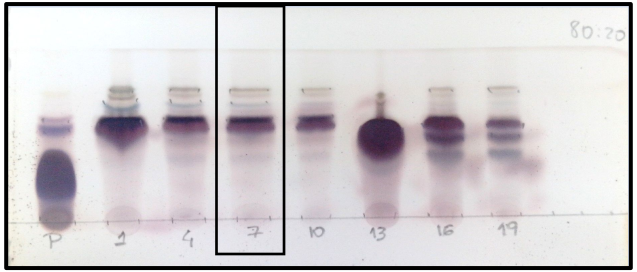

$CH_3OH$

S1C

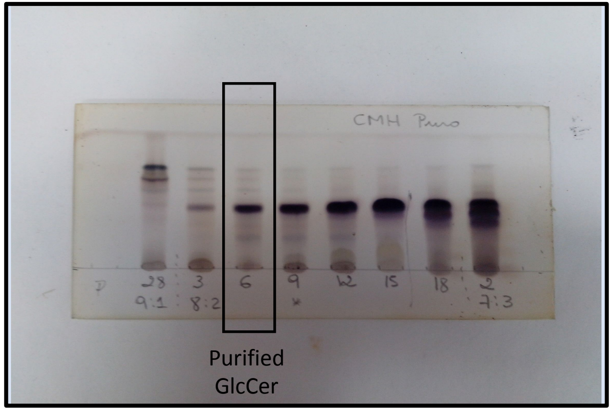

# Supporting information file S1

**Figure S1A.**

**X ↓ X ↓ ↓ X X**

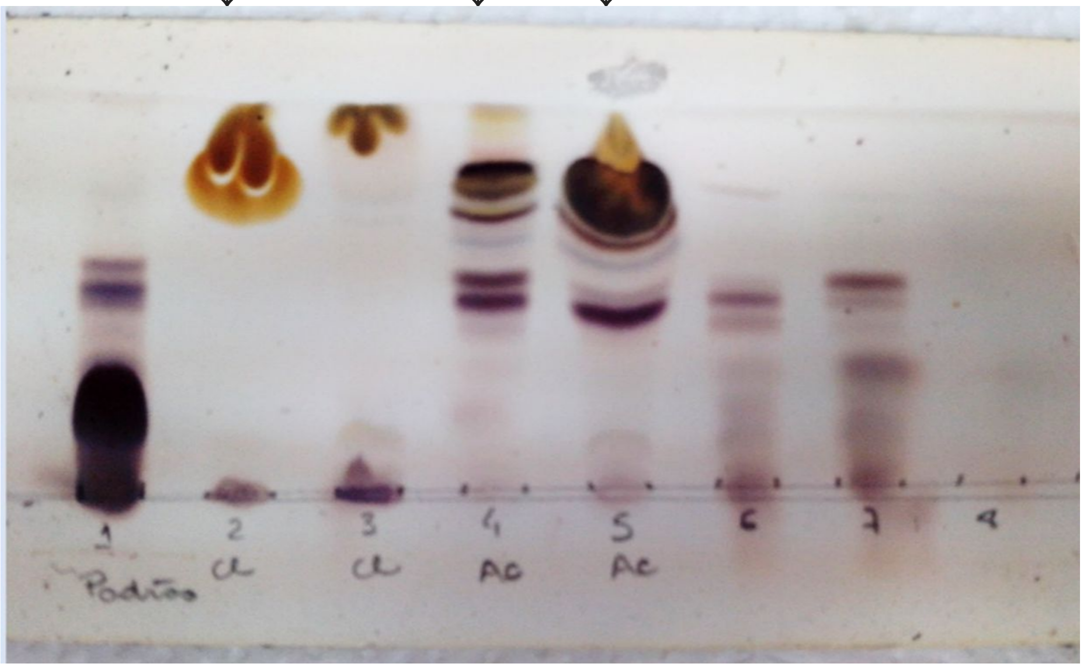


**S1A.** TLC profile of neutral lipid fractions from *F. oxysporum* subjected to silica gel column chromatography and eluted with chloroform, acetone and methanol. Lane 1 - impure GlcCer standard used for comparison. Lanes 2-3 - chloroform fractions. Lanes 4-5 - acetone fractions. Lanes 6-7 - methanol fractions. Solvent system: chloroform/methanol ½ M NH4OH (40:10:1 v/v/v). Detection: iodine and orcinol–sulfuric acid reagents. Impure glucosylceramides were eluted in lanes 4, 5, 6 and 7.

**Images used in Figure 1:**

**Lane 5 (5 Ac) = lower layer image**

**Lane 2 (2 Cl) = $CHCl_3$ image**

**Lane 4 (4 Ac) = $CH_3COCH_3$ image**

**(Lanes 1, 3, 6, 7 and 8 were not used)**

# Supporting information file S1 B

# lane 4 = $CH_3OH$ shown in Figure 1

**Figure S1 B.**

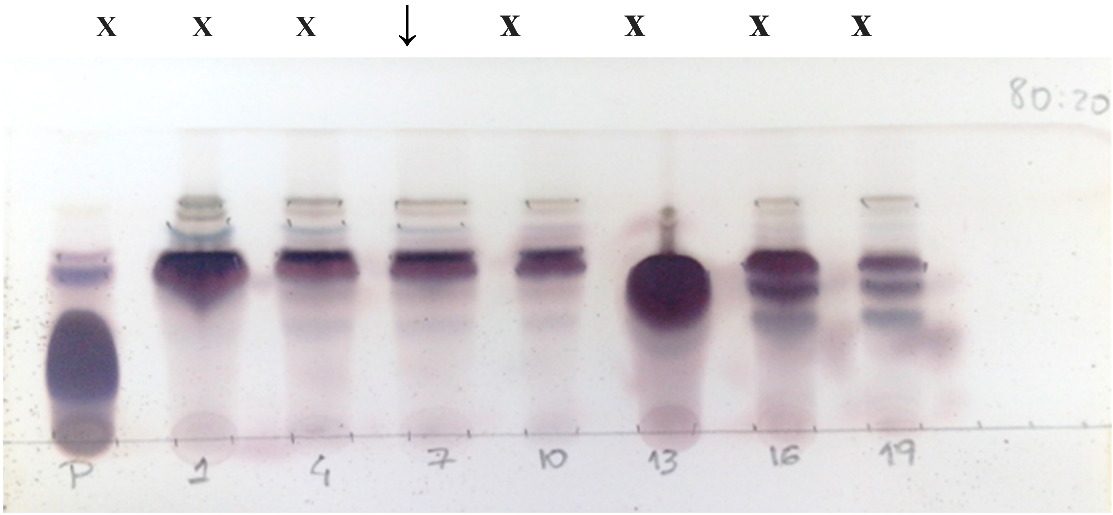


**S1 B.** TLC profile of GlcCer fractions from *F. oxysporum* subjected to silica gel column chromatography and eluted with chloroform/methanol 8:2 v/v (lane 1-19). Solvent system: chloroform/methanol ½ M NH4OH (40:10:1 v/v/v). Detection: iodine and orcinol–sulfuric acid reagents.

**Only lane 4 was used. Please do not consider lanes 1-3 and 5-8.**

# Supporting information file S1 C

# (lane 4 = Figure 1 Purified ClcCer)

**Figure S1C.**

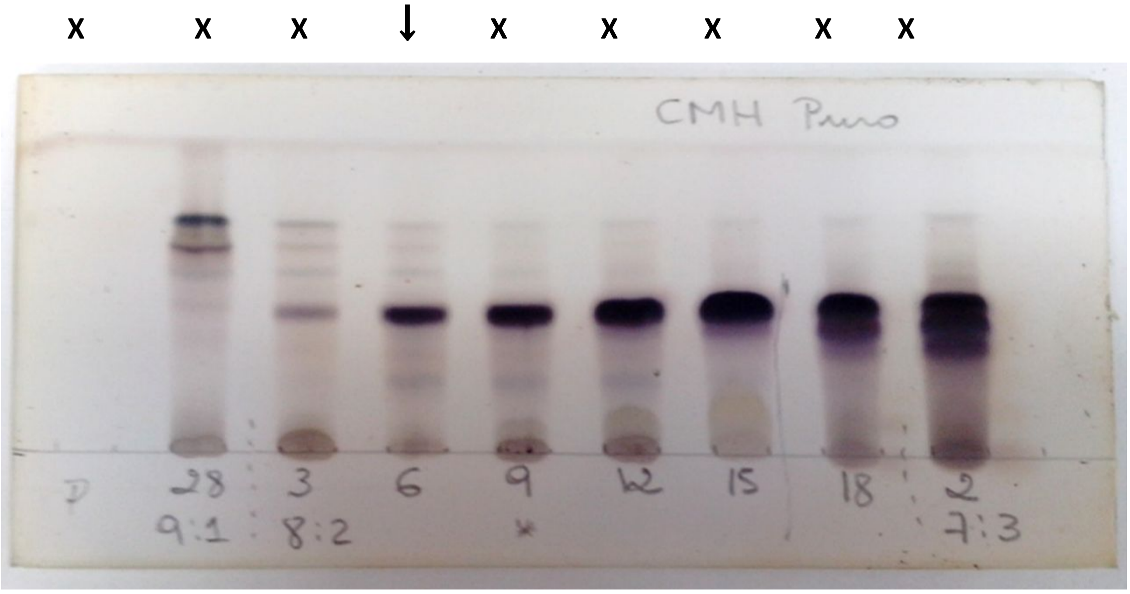


**S1C.** TLC profile of GlcCer fractions from *F. oxysporum* subjected to silica gel column chromatography and eluted with chloroform/methanol 9:1 v/v (lane 28), chloroform/methanol 8:2 v/v (lanes 3-18) and chloroform/methanol 7:3 v/v (lane 2). Solvent system: chloroform/methanol ½ M NH4OH (40:10:1 v/v/v). Detection: iodine and orcinol–sulfuric acid reagents. Purified GlcCer fractions were eluted with chloroform/methanol 8:2 v/v and 7:3 v/v (lanes 18 and 2).

**Only lane 4 was used. Please do not consider any other lane.**

## Supporting information S2: Image used at Figure 2B (bottom part of image is showed at Fig.2B)

**Figure S2.**

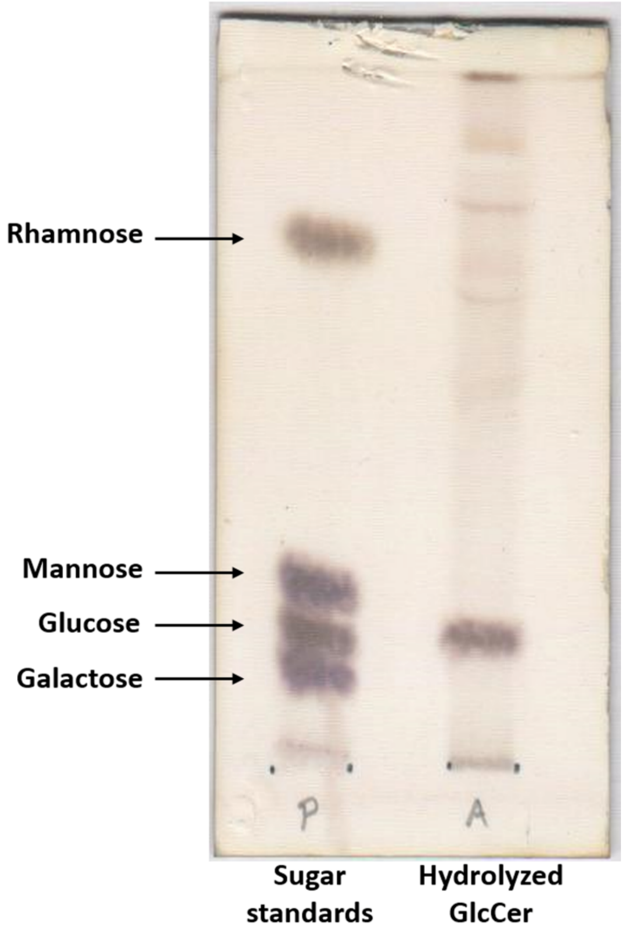


**S2.** Monosaccharide component of GlcCer from *F. oxysporum* characterized by HPTLC in n-butanol-acetone-water (4:5:1v/v). The sugars were detected using an orcinol-sulfuric acid spray reagent. Sugar identification was done by comparison with reference sugars (rhamnose, glucose, mannose and galactose).
